# Supplementary material for: Understanding vegetable farmers’ adoption, dis-adoption, and non-adoption decisions of pest management by pheromone trapping
Source: PLoS One. 2023 Sep 29;18(9):e0292254. doi: 10.1371/journal.pone.0292254 (PMC10540953; doi:10.1371/journal.pone.0292254)
Supplement: Attachment — Submitted filename: PONE-D-22-34820-adoption-nonadoption-pheromone traps.pdf [file pone.0292254.s002.pdf]

PONE-D-2234820

I have read the manuscript with great interest. It is an important piece of work as authors investigate reasons for adoption, disadoption and nonadoption of pheromone trapping for pest control. Their recommendation will benefit Extensions, policy makers and farmers. Indeed, many IPM technologies including mass trapping by pheromone traps are not used by farmers specially in developing countries despite their substantial promotion and training of farmers. So, why this is happening? Authors tried to focus few points of diasadoption and nonadoption in this manuscript. Interesting. However, this very nice piece of research are not well presented, in other words, I felt the manuscript is not well-written. I have provided my section by section suggestion and correction below:

TITLE: I suggest to make it “Understanding vegetable farmers’ adoption, dis-adoption and non-adoption decision of pest management by pheromone trapping”. Later part of the title distract readers. Moreover, pheromone trap is not used for monitoring only rather its suggested to farmers for mass trapping of male insects. This aspect could be mention in the Introduction.

ABSTRACT:

Line (L) 31: “for managing insect pests” can be added after traps for more clarity.

L 32: Rationale of the study are not in the abstract that could be add by putting a sentence only. Please add something like this “Mass trapping by pheromone traps has been promoted and suggested to vegetable farmers of Bangladesh, however, majority of farmers continued insecticide spraying instead of trapping.” The present study investigated……..

L36: What makes them to abandon trapping? Please treat the reasons for not continuing the trapping as next sentence.

L38: Why and how they perceived it was useful? Please be explicit. What was in the interview? Asking simply “ is it useful or not?”. Also, have these farmers made more contact with extension and technical person rather than pesticide company representatives?

L39: In connection to my previous comment this contrast becomes more logical. Is it?

L40: Information and communication technology is a very broad term. Please be precise from your study exactly what information and communication gap you sensed for this non adoption and disadoption.

L41-43: The private sector’s involvement in commercializing………Why do you think so? What have you found from your study?

I have given very detailed comment for Abstract (as an example) as I felt core of this paper are not explicit due to lack of cohesion in story telling. However, this manuscript can be much improved with a little thought and effort in writing. I hope authors will take care that.

INTRODUCTION:

A lot of sentence reshuffling can make the introduction far better. As such its very hard to understand the rationale of the study. For example, as pheromone trap and its adoption issues are the core of the study, it can be treated in first and second paragraph. I understand authors tried to come down local from global aspect, however, global aspect (population increase, vegetable production issues) should be shorter.

L77-82: AVRDC's development of eggplant borer trapping and next to it few sentences on spodolure for cabbage and cotton bollworm. It took quite an effort to understand the story. With reference authors convey that pheromone trapping is an effective method of insect control. Its better to treat those sentences when you introduced general aspect of pheromone trapping in L72. For example, tell about pheromone trap and it has been reported to control variety of insects by using trapping (ref) including eggplant shoot and fruit borer in Bangladesh. Then, what happened to this technology? Is it with farmers and not . Then comes adoption issue . Then rationale of your study. L86-90, please treat with AVRDC issue.

L104-109: Authors indicated novelty of their research. Yes, true there are good papers on IPM adoption issues however there are scanty on pheromone trapping. But this is not novelty of your research. Rather please make explicit that mass trapping by pheromone traps is a nice IPM tools worldwide that can reduce pesticide use substantially and benefit environment. Unfortunately, farmers are not using it. To make this adoptable to farmers, core causes of non and dis-adoption need to be investigated. Until now this has not yet been properly investigated. Then highlight in the conclusion that you left few messages for policy makers to work on pheromone trap adoption. Indeed, many IPM tools are abandoned by farmers probably for same causes you have found for phero trapping. So I think your study also is an example of that.

L110-112: Please shift in conclusion

MATERIALS AND METHODS: Seems okay with few edit.

Please treat ethical consideration in the paragraph of your interview designing.

What kind of interview it was? Structured questionnaire? Semi structured or informal interviews?

Have you categorized the adopter, disadopter and non adopter before interviewing? Or you have selected farmers those have heard about trapping or experienced using it and while interviewing you have understood which farmers falls in which category?

RESULTS: Is okay.

Table 2 foot note b= 247 decimals makes a hectare

L261-64: Fits in materials and methods.

DISCUSSION:

Everything is there without linking each other.

Its better to mention core results and make a discussion on it. For example, those who have high income are non-adopters and they have larger fields. Please discuss why they are non-adopters and how larger fields make them non-adopter?

L308-326: Please make your message clear.

L327-331: In my view, core of your manuscript. Unfortunately it discussed less.

Farmers age, income, size of farming influenced the adoption then why don't you start from socioeconomic causes and then go for labour and time of implementation issues and then source of information, extension training causes and discuss part by part to reach a conclusion. I would suggest start the discussion with summarizing your core results like " Farmers decision on trapping was influenced by age, income, land size….. of farmers). Then slice out one by one in the next few

paragraphs. I find your core is labour and time use in trapping, information from neighbours and exposure to pesticide representatives.

L366-368: Please be explicit what is “appropriate effort” and why private sector commercialization needed, why not government commercialization?

CONCLUSION:

L381-387: Fits better on starting of discussion.

L387: Time consuming nature: Is it pheromone traps act slower than pesticides or it takes time to install and manage the traps? Pls be explicit with suitable words.

You have concluded farmers should be given incentive, unavialbility of pheromone traps and lure should be solved. Please discuss the unavailability issue in discussion section. Please make more clearer in discussion why large farmers abandon this technique compared to small farmers, afterwards please conclude that large farmers need special attention.

All in all, please pick core message to tell in conclusion and highlight on your recommendation and further research too.
